# A Comparison of the Mortuary Statistics of San Francisco, Chicago, Cincinnati, Philadelphia, Charleston, Boston, St. Louis Richmond, Baltimore, New Orleans, New York and Lowell, Mass.

**Published:** 1877-10

**Authors:** E. Fletcher Ingals


### A COMPARISON OF THE MORTUARY STATISTICS OF SAN FRANCISCO, CHICAGO, CINCINNATI, PHILADELPHIA, CHARLESTON, BOSTON, ST. LOUIS, RICHMOND, BALTIMORE, NEW ORLEANS, NEW YORK AND LOWELL, MASS.

By E. Fletcher Ingals, M. D.

From this table we find that the greatest proportion of deaths from phthisis occurs in Lowell, Mass. Next follow Boston and San Francisco at fifteen per cent.; several cities at 11.68 to 14.42 per cent; St. Louis 8.6, and Chicago 7.6 per cent. In these same cities the per centage of deaths from acute pulmonary affection, classed as pneumonia, congestion of the lungs, and bronchitis, is as follows:

Chicago, 7.8 per cent.; St. Louis, 8.3; Charleston, 6.55; New Orleans, 6.22; Cincinnati, 8.9; Philadelphia, 9.17; Richmond, 7.13; Baltimore, 5.85; Boston, 10.49, and San Francisco 8.4 per cent. While these statistics are not absolute proof of the advantages for those predisposed to pulmonary disease, to be derived from a residence in any one of these cities, they show that the dangers to the community from phthisis are much less in Chicago and St. Louis than in the other cities. Grouping together those cities in which the mortality from phthisis is greatest, we find that, with one exception, they are located on or near the sea shore; while those in which it is least are inland. We have not the statistics from Buffalo, but from a table, which is in our possession, we find that the per centage of deaths in that city from consumption is a little lower than in any other city in the United States. From this, it appears that phthisis is more common on the sea coast than inland, and that proximity to a large inland lake, instead of increasing the mortality from this

| | Years | Total Mortality | Deaths from Phthisis and Hæmoptysis | Percentage of Total Mortality | Deaths from Pneumonia, Bronchitis, and Congestion of the Lungs | Percentage of Total Mortality | Deaths from Diseases of the Heart | Percentage of Total Mortality | Deaths from Rheumatism | Percentage of Total Mortality | Deaths from Aneurisms | Percentage of Total Mortality | Deaths from Typhoid and Typho-Malarial Fevers | Percentage of Total Mortality | Deaths from Cholera Infantum, Cholera Morbus Enteritis, Entero-Colitis, Diarrhœa and Dysentery | Percentage of Total Mortality | Deaths from Cholera, Diphtheria, Scarlatina, Cerebro-Spinal Meningitis, and Variola | Percentage of Total Mortality |
|---|---|---|---|---|---|---|---|---|---|---|---|---|---|---|---|---|---|---|
| *San Francisco | 1872–'73, '74 & '75. | 13897 | 2087 | 15. | 1171 | 8.4 | 625 | 4.5 | 35 | .25 | 167 | 1.2 | 412 | 2.96 | 888 | 6.39 | 866 | 6.23 |
| Chicago | 1872–'73, '74 & '75. | 35637 | 2727 | 7.6 | 2790 | 7 6 | 641 | 1.8 | 96 | .27 | 22 | .06 | 1214 | 3.40 | 6618 | 18.57 | 2810 | 7.65 |
| Cincinnati | 1872–'73, '74 & '75. | 21482 | 2638 | 12 28 | 1912 | 8.9 | 533 | 2.48 | 53 | .24 | 15 | .06 | 514 | 2.39 | 1968 | 9.16 | 3456 | 16.08 |
| Philadelphia | 1872–'73, '74 & '75. | 67254 | 9491 | 14.11 | 6173 | 9.17 | 2438 | 3.62 | 173 | .25 | 72 | .10 | 1678 | 2.49 | 6929 | 10.3 | 6074 | 9.03 |
| Charleston, S. C. | 1871–'73, & '75 | 5050 | 590 | 11.68 | 331 | 6.55 | 144 | 2.8 | 12 | .23 | 6 | .11 | 103 | 2.03 | 374 | 7.40 | 236 | 4.67 |
| Boston | 1872–'73, '74 & '75. | 33597 | 5045 | 15.01 | 3525 | 10.49 | 1164 | 3.46 | 165 | .49 | 18 | .05 | 810 | 2.41 | 4156 | 12.37 | 3521 | 10.48 |
| St. Louis | 1872–'73, 74 & '75. | 24136 | 2078 | 8.6 | 2004 | 8.3 | 267 | 1.1 | 49 | .19 | 8 | .03 | 447 | 1.85 | 3343 | 13.85 | 4384 | 18.16 |
| Richmond | 1873 & 1874 | 3628 | 513 | 14.14 | 259 | 7.13 | 122 | 3.36 | 5 | .13 | 1 | .01 | 125 | 3.44 | 478 | 13.17 | ...... | ...... |
| Baltimore | 1873–'74 & '75 | 22605 | 3261 | 14.42 | 1323 | 5.85 | 598 | 2.64 | 160 | .69 | 10 | .04 | 660 | 2.94 | 2790 | 12.34 | 1937 | 8.56 |
| New Orleans | 1873–'74 & 75 | 20420 | 2506 | 12.27 | 1271 | 6.22 | 617 | 3.02 | 27 | .13 | 20 | .09 | 236 | 1.15 | 2299 | 11.25 | 1905 | 9.32 |
| New York | 1874 & '75 | 59436 | 8206 | 13.80 | ...... | ...... | ...... | .... | ... | .... | .... | .... | ...... | .... | four years | 13.75 | ...... | ...... |
| Lowell, Mass | 1874 & '75 | 2201 | 397 | 18.03 | ...... | ...... | ...... | .... | ... | .... | .... | .... | ...... | .... | ...... | ...... | ...... | ...... |

* The Chinese are excluded from the statistics of San Francisco.
* Rubeola was omitted from this list accidentally. There were very few deaths from cholera in any of the cities of this list for any of these years.

disease, seems to lessen it, as shown by comparing the statistics from Buffalo and Chicago with those of St. Louis.

If we find the per centage of deaths from phthisis, to the total mortality, less the number of deaths produced by the principal enteric and zymotic disorders, we obtain the following results:

San Francisco, 17.18 per cent.; Chicago, 10.44 per cent.; Cincinnati, 16.42; Philadelphia, 17.49; Charleston, 13.28; Boston, 19.42; St. Louis, 12.66; Baltimore, 18.24; New Orleans, 15.45. Though the ratio is changed by this computation, still the general results remain the same. As there is only one condition common to San Francisco, Boston, Philadelphia, New Orleans and Baltimore, we have much reason for suspecting that residence near the sea shore is prejudicial to phthisical patients. Yet we cannot set this down as a rule unless we make an exception of Cincinnati. The large mortality from phthisis in San Francisco has been explained by the statement that many consumptives go there to die. This is doubtless true to a certain extent, but it is also true that those who settle new countries are usually uncommonly vigorous persons, or, in other words, those who are least likely to die of phthisis; still this explanation may be in part correct, for we find that before the completion of the railroad, viz., in '66 and '67 the per centage of deaths from consumption and hæmoptysis was only 13.99, whereas for the years '72, '73, '74 and '75 it was 15 per cent. However we cannot place much stress upon this explanation, for it is doubtless true that of the consumptives who leave the older States for the Pacific Coast, in pursuit of health, very few remain in San Francisco, because the ocean winds that daily come in through the Golden Gate drive them south, or inland, to more sheltered places.

As we would naturally suppose, acute pulmonary affections are shown to be less frequent in southern than in northern cities. We observe that in those cities where the per centage of deaths from phthisis is greatest, the per centage of deaths from diseases of the heart is also large, and the ratio remains much the same after deducting the principal enteric and zymotic causes of death. This disfavors the old idea that phthisis, and diseases of the heart are antagonistic; or, at least, it suggests that in some cases they may possibly result from similar causes.

In San Francisco heart diseases cause 4.5 per cent. of the total mortality, while the average mortality in all the other cities from these affections is only 2.79 per cent. This brings us to the most singular fact revealed by this table; viz., the surprising prevalence of aneurisms in California. The mortality from this cause alone in San Francisco amounts to *one hundred and twenty* in every ten thousand deaths; while in all the other cities of this list it is only *nine* in every ten thousand. The principal causes predisposing to aneurisms are said to be 1st. fatty degeneration of the arterial coats; 2d. age (after puberty); 3d. forcible, irregular and occasionally greatly increased action of the heart; 4th. climate; 5th. cachexy, and 6th. obstacles to the free flow of blood through the arteries or capillaries. Of these causes, the second, age, and fifth, cachexy, can have no more to do with the production of aneurisms in San Francisco than in any other city. The first, fatty degeneration, would seem to be equally impotent. The sixth, obstacles to the flow of blood, possibly has some significance, for it may be that all the inhabitants of California are more accustomed to the abuse of alcoholics than those of other portions of the United States, and that as a consequence they are more liable to diseases of the liver and kidneys, which, by causing contraction of the capillaries, lead directly to cardiac disease and dilation of the larger arteries. However we are unwilling to believe that the people of San Francisco are more intemperate than those of many other cities.

The fourth cause enumerated would not favor the production of aneurisms in San Francisco, for it has not a cold climate. It has been stated that aneurisms are more common in Great Britain and Ireland than elsewhere; that they are common in the United States, less common in France and Germany, and rare in the East Indies. This difference Mr. Erichsen attributes to the effects of temperature; but perhaps it might with greater propriety be attributed to the use of alcohol. Certainly, we never in any other place witnessed so much inebriety as in Great Britain. Wines are used freely on the continent of Europe, but we are of the impression that drunkenness is less common there than in our own country.

The third cause enumerated, forcible, irregular, and often excessive action of the heart, is doubtless a potent agent in swelling the per centage of deaths from aneurisms in San Francisco. Probably in no other city in the Union, if in the world, is the excitement of business so great. The mania for speculation, which seems to have fallen upon nearly every man, woman and child in the Golden City, together with the great and almost daily fluctuations in mining and other stocks, must keep the community in a state of excitement which cannot fail to affect the circulatory organs of susceptible individuals. The relaxation of the muscular system, which every one has observed as incidental to sudden changes from a low to a high temperature, may possibly have some influence in the production of aneurisms, and the sudden change from a high to a low temperature, which takes place nearly every summer's day in San Francisco, must cause contraction of the superficial capillaries with obstruction to the flow of blood through them and consequently increased pressure in the larger arteries.

Richmond suffered most from typhoid and typho-malarial fevers, which caused in that city, 3.44 per cent. of its total mortality.

St. Louis suffered most from the principal zymotic disorders which caused 18.16 per cent. of its total mortality. Next to St. Louis in this category, comes Cincinnati, where the per centage was 16.08. Charleston, S. C., suffered least from this class of diseases; viz., 4.67 per cent. From the principal enteric disorders Chicago loses most; viz., 18.57 per cent., and San Francisco least, 6.39. St. Louis loses 13.85 per cent.; New York, 13.75; Richmond 13.17; New Orleans 11.25; Boston, 12.37; Cincinnati, 9.16; Charleston 7.40. This is a matter of special interest to us. Of the 6,618 deaths which occurred in this city during the last four years, 4,969 were from cholera infantum alone. If we could prevent this one disease, or the various affections which are reported under this name, and which whether actually cholera infantum or not, doubtless depend upon similar causes; Chicago would be the healthiest city in the United States. The most influential causes in the production of cholera infantum, are, first, continuous heat for several days at 85°, 95° F; second, unwholesome diet, especially immature or decaying vegetables, and impure milk; and third, those well recognized causes which favor the production, or foster the continuation of epidemic cholera.

Probably there is not a city in this list where the average summer temperature is lower than in Chicago, unless it be San Francisco, and there is certainly not one where wholesome food is cheaper and more abundant. We presume the milk which is supplied to our inhabitants is as pure as that used in other large cities. Therefore, we think neither temperature nor diet can account for the great mortality in this city from cholera infantum.

Authors agree that the most potent causes of this disease are the same as those which favor the production, or continuance and propagation, of epidemic cholera. First and foremost among these are animal poisons arising from the decomposition of organic matter in and about slaughtering houses, glue factories, rendering establishments, &c. Among other causes, are bad drainage, obstructed sewers, neglected privies, overcrowded tenements, and poor ventilation. The latter of these causes cannot be especially operative in Chicago, and probably they are even less active here than in most other cities on this list; for Chicago is built on a broad prairie over which the wind sweeps without obstruction, its inhabitants are not crowded, and they have an inexhaustible supply of pure water. The drainage here may be defective, still it is much better than in New Orleans, where the per centage of deaths from these diseases is only 11.25, and it is doubtless as good as in Charleston, where the percentage is only 7.40. We do not think our large mortality from enteric affections can be due to poor drainage. In St. Louis where the drainage is very perfect, the percentage of deaths from these causes is larger than in New Orleans. The first and most potent cause of cholera infantum and kindred diseases; viz., decomposing organic matter, seems to be the one from which we suffer most, and there seems no opportunity for doubting that we could greatly lessen the mortality from this affection by removing the causes of that noisome stench, which during the warm months, comes up from Brigdeport and the North Branch, and spreads like a putrescent pall all over the entire city.

Summarising the results of this analysis, we have the following conclusions, which, although they cannot be set down as established facts, still bear the stamp of strong possibilities.

1st. Residence by the sea-shore is prejudicial to phthisical patients.

2d. There is something peculiar to the climate of San Francisco, or to the business and social relations of its inhabitants, which strongly predisposes to aneurisms.

3d. A large percentage of the mortality in Chicago is the direct result of organic poisons emanating from the slaughter-houses, glue factories and rendering establishments of Bridgeport, and the foul water in the North Branch.

